# Blood-derived lncRNAs as biomarkers for cancer diagnosis: the Good, the Bad and the Beauty

**DOI:** 10.1038/s41698-022-00283-7

**Published:** 2022-06-21

**Authors:** Cedric Badowski, Bing He, Lana X. Garmire

**Affiliations:** 1 University of Hawaii Cancer Center, Epidemiology, 701 Ilalo Street, Honolulu, HI 96813 USA; 2 Present Address: Department of Computational Medicine and Bioinformatics, University of Michigan, Ann Arbor, MI 48105 USA

**Keywords:** Tumour biomarkers, Cancer screening

## Abstract

Cancer ranks as one of the deadliest diseases worldwide. The high mortality rate associated with cancer is partially due to the lack of reliable early detection methods and/or inaccurate diagnostic tools such as certain protein biomarkers. Cell-free nucleic acids (cfNA) such as circulating long noncoding RNAs (lncRNAs) have been proposed as a new class of potential biomarkers for cancer diagnosis. The reported correlation between the presence of tumors and abnormal levels of lncRNAs in the blood of cancer patients has notably triggered a worldwide interest among clinicians and oncologists who have been actively investigating their potentials as reliable cancer biomarkers. In this report, we review the progress achieved (“the Good”) and challenges encountered (“the Bad”) in the development of circulating lncRNAs as potential biomarkers for early cancer diagnosis. We report and discuss the diagnostic performance of more than 50 different circulating lncRNAs and emphasize their numerous potential clinical applications (“the Beauty”) including therapeutic targets and agents, on top of diagnostic and prognostic capabilities. This review also summarizes the best methods of investigation and provides useful guidelines for clinicians and scientists who desire conducting their own clinical studies on circulating lncRNAs in cancer patients via RT-qPCR or Next Generation Sequencing (NGS).

## Introduction

Cancer ranks as one of the deadliest diseases worldwide. Despite ongoing efforts to develop new treatments and a better understanding of the mechanisms underlying tumorigenesis, it remains difficult to treat cancers, particularly when diagnosed at late stages with a poor prognosis. The high mortality rate associated with cancer is partially due to the lack of early detection methods and/or inaccurate diagnostic tools, such as certain protein biomarkers. Protein or peptide-based biomolecules such as glycoproteins constitute most of the currently available cancer biomarkers. Variations in their levels in tissues or blood may indicate the development of diseases such as cancer. Protein markers can be detected in tissue biopsy sections analyzed by immunohistochemistry (IHC) upon diagnostic notably to determine cancer molecular subtype. For instance, breast tumor tissues are commonly assessed for the presence of estrogen receptor (ER) to determine their ER-positive or ER-negative status. However, some protein biomarkers are reportedly unreliable as they generate a significant amount of false-positive and/or false-negative results. Plasma alpha fetoprotein (AFP), one of the most frequently used biomarkers for diagnosis of hepatocellular carcinoma (HCC)^1^, has been described by many as a marker with low sensitivity and/or specificity^2–5^. Conventional serological biomarkers such as carbohydrate antigen 153 (CA153), cancer antigen 125 (CA125), CA27.29 and carcinoembryonic antigen (CEA) remain controversial due to poor specificity and sensitivity^6–11^. The poor reliability of certain protein biomarkers is partially due to the nature of the biomarker itself. The detection of proteins and peptides indeed relies on the use of antibodies that may or may not be specific to the desired marker as the epitope recognized by the antibodies may be present on other tissue components^12^. Unreliable antibodies currently represent a major issue in biomedical research in general and can significantly comprise the outcome of a study or diagnosis. Another issue with traditional histology analyses is the need for actual tissue biopsies. This invasive and inconvenient technique may discourage potential cancer patients to proceed with the entire diagnosis procedure. Thus, the development of noninvasive nonprotein biomarkers is currently needed.

Cell-free nucleic acids (cfNA) or circulating nucleic acids (CNAs) have recently been proposed as a new class of potential biomarkers that could improve cancer diagnosis^13^. CNA PCA3 (prostate cancer associated 3) has notably been approved by the FDA and is currently being sold as Progensa by Hologic Gen Probe (Marlborough, MA, USA) for the diagnosis of prostate cancer^14–16^. Circulating long noncoding RNAs or lncRNAs (noncoding RNAs of 200 nucleotides or more), such as PCA3 seem more reliable than other CNAs due to their high stability in the bloodstream and poor sensitivity to nuclease-mediated degradation. Arita et al. especially showed that plasmatic lncRNAs are resistant to degradation induced by repetitive freeze-thaw cycles, as well as prolonged exposure to 45 C and room temperatures^17^. The stability of lncRNAs in the bloodstream appears to originate from the presence of extensive secondary structures^18^, the transport by protective exosomes^19^, as well as stabilizing posttranslational modifications. The reported prevalence of ncRNAs in the mammalian genome and the known association between aberrant lncRNA expressions and tumorigenesis undeniably highlight the crucial biological importance of ncRNAs in health and disease. NcRNAs are particularly known to be major regulators of cell proliferation and differentiation during development and in adult life through complex mechanisms which are still being investigated. In a pioneering study published in 2007, Rinn et al. notably reported that lncRNA HOTAIR (HOX transcript antisense RNA) was capable of repressing transcription in trans across the HOXD locus and interacting with Polycomb Repressive Complex 2 (PRC2) while being required for PRC2 occupancy and histone H3 lysine-27 trimethylation of HOXD locus^20^. Many more mechanisms have been described and continue to be discovered, as scientists and clinicians actively investigate the mechanisms of action of lncRNAs as well as their potential as reliable cancer biomarkers.

The high stability and relative abundance of lncRNAs in the circulation may make them more reliable cancer biomarkers compared to other analytes such as circulating tumor cells (CTCs), cell-free DNA (cfDNA, which includes circulating tumor DNA ctDNA) and exosomes. CTCs and ctDNA are present in limited quantities in the fluids of cancer patients especially those with early-stage cancers, which may significantly hinder their quantification in clinic, while impairing the detection of low allelic frequency mutations^21,22^. Moreover, CTCs are very heterogenous^21^, and the value of CTCs as diagnostic biomarkers remains currently unclear as early lesions may still be benign and devoid of CTCs^21^. ctDNA on the other hand, may not be sufficient to provide an accurate diagnosis and is often used in combination with other methods in diagnostic and prognostic studies. As for tumor-derived exosomes, the detection of glycoprotein biomarkers on their surface relies heavily on the specificity of antibodies. Lysed exosomes could be alternatives that release the nonprotein content including lncRNAs, which are easier to detect compared to proteins.

In this report, we review the progress achieved and challenges encountered in the development of circulating lncRNAs as potential biomarkers for early cancer diagnosis. We report and discuss the specificity and sensitivity of blood-based lncRNAs currently considered as promising biomarkers for various cancers such as hepatocellular carcinoma, colorectal cancer, gastric cancer and prostate cancer. We also highlight potential therapeutic applications for circulating lncRNAs both as therapeutic targets and agents, on top of diagnostic and prognostic purposes. Based on recommendations from different published works, we finally provide recommendations for investigators who seek to investigate and compare the levels of circulating lncRNAs in the blood of cancer patients compared to healthy subjects by RT-qPCR or Next Generation Sequencing.

## Blood-based lncRNAs as potential circulating biomarkers for cancer diagnosis

### Changes in circulating lncRNA levels specifically correlate with cancer development

Most studies focusing on circulating lincRNAs have been initiated based on prior observations reporting changes in lncRNA levels in cancer tissue samples. For instance, MALAT-1 (metastasis-associated lung adenocarcinoma transcript 1) was first shown to be upregulated in various cancer tissues including lung and prostate tumors^23,24^. Using peripheral blood cells as a lincRNA source for their study, Weber et al. later showed that MALAT-1 levels could reflect the presence of nonsmall-cell lung cancer with a specificity of 96%^25^ (Table 1). LncRNA MALAT-1 was also detected in significantly higher quantities in the plasma of patients with prostate cancer as compared to healthy subjects^26^ and these changes in circulating MALAT-1 levels correlated with prostate cancer with relatively high specificity (84.8%)^26^. This study showed that tumors were at the origin of MALAT-1 variations, since the surgical removal of the cancerous tissues induced a dramatic reduction in circulating MALAT-1, while plasmatic levels of this lncRNA increased upon ectopic implantation of a tumoral xenograft in mice^26^. More studies support the concept that circulating lncRNAs are, directly or indirectly, correlated with the presence of tumors in vivo. For instance, the blood of patients with hepatocellular carcinoma was shown to contain elevated levels of lncRNA HULC (for “highly upregulated in liver cancer”)^27,28^. Moreover, HULC, H19, HOTAIR and GACAT2 (for “gastric cancer-associated transcript 2”) were found to be significantly increased in the plasma of gastric cancer (GC) patients compared to healthy individuals^29–32^. Alike MALAT-1 which was primarily detected in tumoral tissue, lncRNA GIHCG (for “gradually increased during hepatocarcinogenesis”) was originally found to be upregulated in cancer tissue samples from HCC and RCC (renal cell carcinoma) tumors^33,34^. Higher levels of GIHCG as well as ARSR (for “activated in RCC with sunitinib resistance”) were also reported in the circulation of renal cell carcinoma patients^34–36^. Serum GIHCG levels were notably able to distinguish RCC patients from healthy individuals with a specificity of 84.8%. Levels of circulating lncRNAs GIHCG and ARSR significantly dropped after resection of RCC tumors, while plasma levels of H19, A174084 and GACAT2 markedly decreased in GC patients postoperatively, further supporting a direct correlation between abnormal levels of circulating lncRNAs and tumorigenesis^29,32,34,35,37,38^. In fact, some of these circulating lncRNAs have shown greater diagnostic performance than conventional glycoprotein markers. For instance, circulating H19 and RP11-445H22.4 have been reported as more reliable than carcinoembryonic antigen (CEA) and/or carbohydrate antigen 153 (CA153) for the diagnosis of breast cancer^39,40^. Likewise, a serum three-lncRNA signature consisting of PTENP1, LSINCT-5 and CUDR (also known as UCA1) significantly outperformed CEA and CA19-9 in gastric cancer diagnostic studies^41^.

**Table 1:** List of blood-based lncRNAs investigated as potential biomarkers for diagnosis of various cancers.

LncRNA	Cancer type	Source	Sensitivity (%)	Specificity (%)	AUC / QUADAS	Normalization	Reference
MALAT-1	Nonsmall-cell lung cancer	Blood cells	56	96	AUC 0.79	GAPDH	^25^
	Nonsmall-cell lung cancer	Whole blood	N.A.	N.A.	AUC 0.718	GAPDH	^51^
	Prostate cancer	Plasma	58.6	84.8	AUC 0.836	Standard curve	^26^
	Hepatocellular carcinoma	Plasma	51.1	89.3	AUC 0.66	MALAT-1	^48^
LINC00152	Gastric cancer	Plasma	48.1	85.2	AUC 0.675	GAPDH	^19^
	Hepatocellular carcinoma	Plasma	N.A.	N.A.	AUC 0.85	5 S	^43^
	Hepatocellular carcinoma	Serum	78.3	89.2	AUC 0.877	GAPDH	^44^
UCA1	Hepatocellular carcinoma	Serum	92.7	82.1	AUC 0.861	GAPDH	^45^
	Hepatocellular carcinoma	Serum	91.4	88.6	QUADAS 11	β-actin	^46^
	Colorectal cancer	Plasma	N.A.	N.A.	N.A.	Cel-miR-39	^178^
	Gastric cancer	Plasma	N.A.	N.A.	AUC 0.928	GAPDH	^104^
	Osteosarcoma	Serum	N.A.	N.A.	AUC 0.831	GAPDH	^179^
H19	Gastric cancer	Plasma	74	58	AUC 0.64	LncRNA levels	^17^
	Gastric cancer	Plasma	82.9	72.9	AUC 0.838	Standard curve	^38^
	Gastric cancer	Plasma	68.75	56.67	AUC 0.724	GAPDH	^60^
	Breast cancer	Plasma	56.7	86.7	AUC 0.81	β-actin	^39^
PVT1	Cervical cancer	Serum	71.6	98.8	AUC 0.932	GAPDH	^176^
	Melanoma	Serum	94.12	85.11	AUC 0.938	GAPDH	^177^
WRAP53	Hepatocellular carcinoma	Serum	85.4	82.1	AUC 0.896	GAPDH	^45^
HULC	Hepatocellular carcinoma	Blood cells	N.A.	N.A.	N.A.	β-actin	^27^
	Hepatocellular carcinoma	Plasma	N.A.	N.A.	N.A.	GAPDH	^28^
	Hepatocellular carcinoma	Plasma	N.A.	N.A.	AUC 0.78	5 S	^43^
	Gastric cancer	Plasma	58	80	AUC 0.65	GAPDH	^30^
HOTAIR	Colorectal cancer	Blood cells	67	92.5	AUC 0.87	PPIA	^42^
	Cervical cancer	Serum	N.A.	N.A.	N.A.	GAPDH	^105^
CTBP	Hepatocellular carcinoma	Serum	91	88.5	QUADAS 11	β-actin	^46^
GIHCG	Renal cell carcinoma	Serum	87	84.8	AUC 0.920	N.A.	^34^
	Cervical cancer	Serum	88.7	87.5	AUC 0.940	β-actin	^90^
PCA3	Prostate cancer	Periph. Blood	32	94	N.A.	N.A.	^14^
RP11-445H22.4	Breast cancer	Serum	92	74	AUC 0.904	U6	^40^
uc003wbd	Hepatocellular carcinoma	Serum	N.A.	N.A.	AUC 0.86	β-actin	^49^
AF085935	Hepatocellular carcinoma	Serum	N.A.	N.A.	AUC 0.96	β-actin	^49^
GACAT2	Gastric cancer	Plasma	87	28	AUC 0.622	GAPDH	^29^
SPRY4-IT1	Hepatocellular carcinoma	Plasma	87.3	50	QUADAS 12	18 S	^47^
uc001ncr	Hepatocellular carcinoma	Serum	N.A.	N.A.	AUC 0.885	GAPDH	^50^
AX800134	Hepatocellular carcinoma	Serum	N.A.	N.A.	AUC 0.925	GAPDH	^50^
ZNFX1-AS1	Gastric cancer	Plasma	84	68	AUC 0.85	GAPDH	^30^
LINC00152 + AFP	Hepatocellular carcinoma	Serum	85.3	83.4	AUC 0.906	GAPDH	^44^
XIST + HIF1A-AS1	Nonsmall-cell lung cancer	Serum	N.A.	N.A.	AUC 0.931	GAPDH	^62^
PVT1 + uc002mbe.2	Hepatocellular carcinoma	Serum	60.5	90.6	QUADAS 11	GAPDH	^64^
GAS5 + SRA	Pancreatic cancer (IPMN)	Plasma	82	59	AUC 0.729	β-actin PGK1 PPIB	^69^
SPRY4-IT1 + ANRIL + NEAT1	Nonsmall-cell lung cancer	Plasma	82.8	92.3	AUC 0.876	N.A.	^61^
LINC00152 + UCA1 + AFP	Hepatocellular carcinoma	Serum	82.9	88.2	AUC 0.912	GAPDH	^44^
CUDR (UCA1) + LSINCT-5 + PTENP1	Gastric cancer	Serum	81.8	85.2	AUC 0.829	β-actin	^41^
SPRY4-IT1 + POU3F3 + HNF1A-AS1	Esophageal squamous cell carcinoma	Plasma	72.8	89.4	AUC 0.842	GAPDH	^63^
XLOC_006844 + LOC152578 + XLOC_000303	Colorectal cancer	Plasma	80	84	AUC 0.975	N.A.	^65^
RP11-160H22.5 + XLOC_014172 + LOC149086	Hepatocellular carcinoma	Plasma	82	73	AUC 0.896	β-actin	^3^
UCA1 + POU3F3 ESCCAL-1 + PEG10	Esophageal squamous cell carcinoma	Serum	80.2	80.2	AUC 0.853	GAPDH	^66^
LET + PVT1 + PANDAR + PTENP1 + linc00963	Renal cell carcinoma	Serum	67.6	91.4	AUC 0.823	β-actin	^68^
AOC4P + BANCR + CCAT2 + LINC00857 + TINCR	Gastric cancer	Plasma	0.82	0.87	AUC 0.91	GAPDH	^67^

N.A. not available / data presented in graphical format in original report.

Information reported includes the name of lncRNA, cancer type, source of lncRNA, lncRNA specificity, lncRNA sensitivity, AUC (ROC) value (area under the ROC curve - receiver operating characteristic), QUADAS score, normalization method and literature reference.

Other lncRNAs have been reported to detect various cancer types with relatively high specificity. For instance, HOTAIR has shown high efficacy in identifying samples from colorectal cancer patients with a specificity of 92.5%^42^. Changes in plasmatic levels of lncRNA LINC00152 were found to correlate with gastric cancer with a specificity of 85.2%^19^ (Table 1). LNC00152 has also been suggested as a reliable blood-based biomarker for hepatocellular carcinoma^43,44^. The high prevalence of HCC in certain parts of the world such as Asia or Africa is undeniably alarming, and it has become a major public health matter in many countries. Reliable biomarkers are desperately needed to detect this deadly cancer at an early stage. Many circulating lncRNAs have shown a significant correlation with HCC and represent promising candidates for HCC diagnostic applications (Table 1). Several studies from Egypt identified lncRNA-UCA1 as a potential serum-based biomarker for the detection of HCC. The specificities obtained were 82.1%^45^ and 88.6%^46^. These studies also reported WRAP53 and CTBP as potential biomarkers for HCC with a specificity of 82.1%^45^ and 88.5%^46^, respectively. In Asia, Jing et al. showed that lncRNA SPRY4-IT1 represents another promising blood-based biomarker for the diagnosis of hepatocellular carcinoma^47^.

Many more circulating lncRNAs have been proposed as potential blood-based biomarkers for cancer diagnosis, some with relatively high specificity (Table 1)^48–50^.

### Challenges and potential impacts on diagnosis using lncRNA as biomarkers

The diagnostic power of circulating biomarkers has yet to reach its maximum potential. Indeed, the diagnostic performance of many circulating lncRNAs remains relatively poor when taken individually. Several lncRNAs reportedly have either poor sensitivity or poor specificity towards a specific cancer type, affecting their potentials as diagnosis biomarkers. Below are some examples:

MALAT-1 has shown a sensitivity of only 58,6% when testing plasma samples from prostate cancer patients and healthy subjects. This moderate sensitivity implies that the use of MALAT-1 as a blood-based prostate cancer biomarker may result in a significant number of false-negative results, as actual cancer samples may not be detected. MALAT-1 has also been investigated as a potential biomarker for nonsmall-cell lung cancer^25,51^. However, with a sensitivity of only 56%, MALAT-1 may also face multiple challenges before becoming a reliable blood-based biomarker for lung cancer diagnosis (Table 1). One unsolved issue is notably the reported lack of correlation between the levels of circulating MALAT-1 in lung cancer patients and the levels of this lncRNA in lung cancer tissues. Indeed, the comparative analysis of whole blood samples from 105 lung cancer patients and 65 healthy subjects revealed a decrease in blood MALAT-1 levels in cancer patients, while lung cancer tissues showed higher MALAT1 expression^51^. The lack of strong sensitivity and the poor correlation between tissue and blood levels may arise from the fact that MALAT-1 is reportedly undergoing a certain degree of degradation in the bloodstream^26^. One of the resulting fragments has notably been referred to as MD-mini RNA (for metastasis associated in lung adenocarcinoma transcript 1 derived miniRNA)^26^.

The degradation of MALAT-1 in the bloodstream may not be an isolated case and, probably, many more lncRNAs are actively being degraded once they enter the circulation. Degradation of circulating lncRNAs may increase in cancer patients as several studies reported that tumorigenesis is often associated with higher RNAse activity in the bloodstream^52^. In fact, long before circulating lncRNAs were considered as potential cancer biomarkers, increased RNAse activity in the serum of cancer patients was suggested as a mean of early cancer detection^53,54^. In their study, Reddi and Holland notably reported that 90% of the patients with pancreatic cancer showed a dramatic increase in serum RNAse levels (above 250 units/mL). They hence promoted the use of high serum RNAse activity as a biomarker for pancreatic carcinoma. Other cancers such as chronic myeloid leukemia have also been reported to be associated with a higher level of plasmatic RNAse activity^55^. RNAses circulating in the bloodstream notably constitute cytotoxic agents secreted by immune cells as part of anti-cancer defense mechanisms that aim at lysing transformed cells by activating cell death pathways^56^. For instance, an RNAse secreted by human eosinophils is known to induce the specific apoptosis of Kaposi’s sarcoma cells without affecting normal human fibroblasts^57^. RNAse L was shown to suppress prostate tumorigenesis by initiating a cellular stress response that leads to cancer cell apoptosis^58,59^. Tumors, on the other hand, reportedly display lower RNAse activity to promote protein synthesis and cell proliferation^52^. The reported difference in RNAse activity in tumors versus circulation may explain seemly paradoxical data when comparing lncRNA levels in tissues and blood such as in the case of MALAT1. While many studies have shown positive correlations between tissue and blood lncRNAs, the reported increased RNAse activity in the blood of some cancer patients may promote the degradation of circulating lncRNAs to a degree that would depend on the nature of cancer and/or lncRNA studied. This could represent a significant challenge for investigators as RT-qPCR analyses may not detect fragments of an investigated lncRNA possibly compromising the outcome of a study.

LINC00152 is another circulating lncRNA that has been actively investigated as a potential cancer biomarker. However, LINC00152 has shown a sensitivity of only 48.1% when analyzing plasma samples from gastric patients and healthy subjects, limiting its diagnostic performance as well (Table 1). It is currently not clear if LINC00152 is undergoing degradation in the bloodstream. Other circulating lncRNAs have shown poor specificity in the detection of specific cancers. For instance, GACAT2 reportedly has a specificity of only 28% when comparing plasma samples from gastric cancer patients and healthy subjects^29^, while several studies have shown that H19 is capable of detecting samples from gastric cancer patients with a specificity of only 58 %^17^ or 56.67%^60^ (Table 1). This implies that diagnosis based on the quantification of plasmatic levels of H19 or GACAT2 may potentially result in a significant number of false-positive results when testing for gastric cancer. It is also the case for lncRNA SPRY4-IT1 regarding the diagnosis of hepatocellular carcinoma (HCC) with a specificity of only 50%, and HULC for the detection of gastric cancer (with a specificity of only 58%)^30^ (Table 1).

Therefore, significant improvements are required before most individual circulating lncRNAs become reliable blood-based cancer biomarkers.

### Combination of circulating lncRNAs for greater diagnostic performance and new technologies for improved lncRNA detection

To compensate for the moderate specificity/sensitivity of certain circulating lncRNAs and increase their diagnostic performance, several studies have combined the diagnostic values of several circulating lncRNAs. For instance, Hu et al., integrated lncRNAs SPRY4-IT1, ANRIL and NEAT1 in their studies on nonsmall-cell lung cancer and obtained a specificity of 92.3%, a sensitivity of 82.8%, and an AUC (ROC) (area under the ROC curve - receiver operating characteristic) of 0.876^61^ (Table 1). The combination of serum XIST and HIF1A-AS1 was able to accurately detect nonsmall-cell lung cancer as well^62^. When combined with POU3F3 and HNF1AAS1, SPRY4-IT1 displayed a sensitivity of 72.8% and a specificity of 89,4% (AUC: 0.842) in the detection of esophageal squamous cell carcinoma^63^. Yu et al. reported that the combination of circulating lncRNAs PVT1 and uc002mbe.2 reflected the presence of hepatocellular carcinoma with a specificity of 90.6% and a sensitivity of 60.5%^64^. The integrated analysis of plasmatic levels of XLOC_006844, LOC152578 and XLOC_000303 allowed the detection of colorectal cancer with a specificity of 84%, a sensitivity of 80% and an AUC of 0.975^65^. Other examples include the combination of lncRNAs RP11-160H22.5, XLOC_014172 and LOC149086 which produced a sensitivity of 82% and a specificity of 73% (AUC: 0.896) for the diagnosis of hepatocellular carcinoma^3^ (Table 1). Some studies have investigated the diagnostic signature of more than 3 circulating lncRNAs. For instance, Yan et al, reported that a 4-lncRNA panel comprising UCA1, POU3F3, ESCCAL-1 and PEG10 constitutes a remarkable diagnostic tool for the accurate and reliable detection of esophageal squamous cell carcinoma (ESCC) since this multi-lncRNA panel was capable of distinguishing ESCC patients from healthy controls with a sensitivity of 80.20%, a specificity of 80.20% and an AUC of 0.853^66^. The authors emphasized that, in terms of diagnostic performance, the 4-lncRNA panel outperformed each individual lncRNA, further supporting the clinical value of such a combinatory approach. In a separate study, Zhang et al. identified a panel of five plasma lncRNAs (BANCR, AOC4P, TINCR, CCAT2 and LINC00857) that was able to discriminate GC patients from healthy controls with an AUC of 0.91, outperforming CEA biomarker^67^. Wu et al. have reported that a 5-lncRNA signature could accurately distinguish serum samples of patients with renal cell carcinoma (RCC) from those of healthy subjects^68^. The combination of lncRNA-LET, PVT1, PANDAR, PTENP1 and linc00963 identified RCC samples with an AUC of 0.823. Each of these 5 lncRNAs was not individually capable of performing as well as the 5-lncRNA signature. PVT1 and PANDAR have also been investigated as part of a 8-lncRNA signature in plasma samples of patients with pancreatic ductal adenocarcinoma^69^. The 8-lncRNA signature was identified by using a custom nCounter Expression Assay (Nanostring Technologies, USA) that allows multiplex qPCR analyses using TaqMan probes. A better diagnostic performance may also be obtained through the improved detection of lncRNAs in human samples and novel highly sensitive methods have been recently developed to achieve this purpose. In a remarkable study, Chen et al. recently developed a novel biocompatible electrochemical biosensor referred to as “SPCE Au NCs/MWCNT-NH2” for the ultrasensitive detection of lncRNA MALAT1 in non‑small cell lung cancer^70^. Importantly, the authors highlighted that, compared to traditional RT-PCR, this new method presents several major advantages including faster detection and lower cost while being simpler to operate. In another outstanding study, Morlion et al. developed a unique custom lncRNA capture sequencing approach that relies on a set of 565,878 capture probes for 49,372 human lncRNA genes and which is reportedly capable of enhancing detection sensitivity^71^. This custom enrichment approach achieved major advancements in lncRNA detection, since it enables the detection of a broad repertoire of lncRNAs with better reproducibility and higher coverage than classic total RNA-sequencing methods.

Overall, the signature generated by the combination of several blood-based lncRNAs reportedly provides better diagnostic performance than most individual circulating lncRNAs, while the emergence of new technologies paves the way for a better detection of lncRNAs in human biofluids.

### Circulating lncRNAs as potential blood-based biomarkers for cancer prognosis

Besides being potential blood-based biomarkers for early cancer diagnosis, circulating lncRNAs may also constitute valuable prognosis markers. Most studies assessing the ability of lncRNAs to predict disease evolution and eventual clinical outcome have been performed on cancer tissue samples^72–74^. However, a few studies based on the analysis of blood-derived samples indicate that circulating lncRNAs may also be able to reflect cancer prognosis. For instance, changes in plasmatic levels of lncRNAs XLOC_014172 and LOC149086 can distinguish metastatic HCC from non-metastatic HCC with a specificity of 90%, a sensitivity of 91% and an AUC of 0.934 (combined)^3^. HOTAIR can also be used as a negative prognostic marker for colorectal cancer with a sensitivity of 92,5%, a specificity of 67% and an AUC of 0.87^42^. Moreover, lncRNA GIHCG has been proposed as a potential prognostic biomarker for renal cell carcinoma^34^. The 5-lncRNA signature reported by Wu et al., was also capable of discriminating benign renal tumors from metastatic renal cell carcinoma^68^. Similarly, the 8-lncRNA signature recently described by Permuth et al., reportedly distinguished indolent (benign) intraductal papillary mucinous neoplasms (IPMNs) from aggressive (malignant) IPMNs^69^. This 8-lncRNA-signature reportedly had greater accuracy than standard clinical and radiological features. It was further improved when combined with plasma miRNA data and quantitative radiomic imaging.

While early studies suggest that the analysis of circulating lncRNA levels may contribute to the evaluation of disease progression, more investigations focusing on blood-based lncRNAs are needed to truly appreciate the prognosis power of circulating lncRNAs. The best diagnostic/prognostic performance may actually emerge from the integration of several analytic methods that combine circulating lncRNA data, miRNA data, clinical data, quantitative imaging features^69^ and/or conventional glycoprotein antigens such as carcinoembryonic antigen (CEA)^60^ or prostate-specific antigen (PSA)^14^.

### Circulating lncRNAs as potential therapeutic agents/targets for cancer treatment

**Fig. 1:**
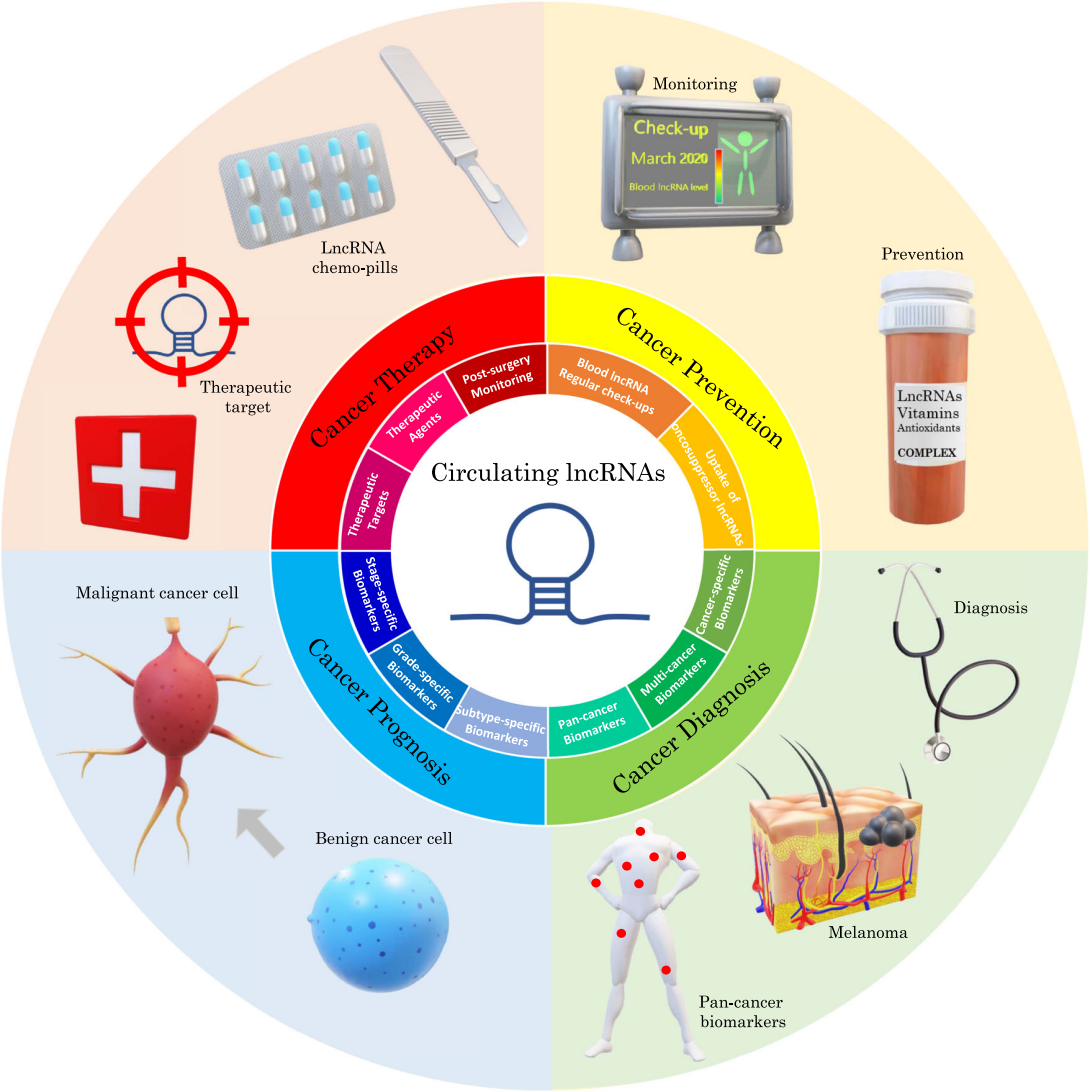
Diagram summarizing the full panel of possible clinical applications that can be derived from the analysis of blood-based lncRNAs. Information indicated includes four main domains of applications (cancer prevention, cancer diagnosis, cancer prognosis, cancer treatment) and smaller subdomains referring to the domain of the same color.

Circulating lncRNAs should not be considered only as passive biomedical tools that solely enable the detection and monitoring of various diseases. They may also constitute effective therapeutic agents and/or targets in innovative strategies that could treat various types of cancers including colorectal cancer and renal cell carcinoma^34,75–77^. Indeed, lncRNAs have been shown to trigger or contribute to tumorigenesis notably by interfering with tumor-suppressive signaling pathways or acting as oncogenic stimuli^78–82^. In a Genome-wide analysis of the human p53 transcriptional network, Sanchez et al. notably revealed the existence of a lncRNA tumor suppressor signature^83^. GAS5, CCND1, LET, PTENP1 and lincRNA-p21 have been described as tumor suppressors^36,75,84–87^, while MALAT-1, PANDAR, HOTAIR, H19, PVT1, GIHCG and ANRIL have been characterized as oncogenic lncRNAs^36,75,88–90^. At the molecular level, lncRNAs can promote tumorigenesis by acting as chromatin structure regulators that modify gene expression^91^, scaffolds for oncogenic RNA-binding proteins^92^ or RNA sponges for oncosuppressor microRNAs^93,94^. For instance, lncRNA HOTTIP (HOXA transcript at the distal tip) was shown to act as a sponge for the tumor-suppressive microRNA miR-615-3p and dysregulation of HOTTIP expression was shown to alter levels of miR-615-3p and its target IGF-2, promoting the formation of RCC tumors^94^. Many more mechanisms have been described and continue to be discovered. Through various pathways, dysregulation of lncRNAs levels eventually promotes cancer cell proliferation, migration, invasion and/or metastasis^94–97^. Therefore, lncRNAs do constitute legitimate therapeutic targets. However, most mechanistic studies have been done on cancer tissues or cells, so it is still unclear if targeting lncRNAs in blood would be sufficient to treat tumors located deep inside layers of tissues. A more fundamental question may be to determine whether circulating lncRNAs can actually penetrate cells and tissues. Nucleic acids are usually unable to cross the hydrophobic cellular plasma membrane due to their large size and negative charges carried by the phosphate groups of nucleotides. In vitro DNA transfection is usually achieved by using specific carriers such as lipofectamine. Answers may come from reports indicating that circulating lncRNAs are, at least for a part, transported in the blood via extracellular vesicles such as exosomes^19^. It has even been reported that 3.36 % of the total exosomal RNA content is represented by lncRNAs^98^. Circulating exosomes are lipid-based extracellular vesicles that promote the transport of various biomolecules across long distances within the human body. Microvesicles and exosomes have notably been characterized as potent messengers that enable cancer cells to communicate with each other (autocrine messengers) and also with non-cancerous cells (paracrine and endocrine messengers^99^. Because of their lipidic structure, exosomes can fuse with the plasma membrane of a targeted cell and release their content inside it, including lncRNAs. It is thus conceivable that exosome-borne lincRNAs may be used by cancer cells to spread within the human body. Therefore, circulating lincRNAs may constitute bonafide therapeutic targets as much as tissue lncRNAs do (Fig. 1). Besides exosomes, some circulating lncRNAs may be transported as complexes with circulatory proteins such as Argonaute (Ago) or nucleophosmin 1 (NPM1) similar to circulating miRNAs^100,101^. Others may be transported in blood without any binding partner or specific protective structure. These lncRNAs may constitute the easiest targets for lncRNA-interfering cancer therapy. While the circulatory system is devoid of cellular machinery that degrades RNA-RNA and RNA-DNA hybrids, targeting lncRNAs using ASOs (RNAseH-dependent antisense oligonucleotide) can effectively produce significant antitumoral effects in vivo. Arun et al. have notably shown that the systemic knockdown of Malat-1 by subcutaneous injections of ASOs in an MMTV-PyMT mouse mammary carcinoma model resulted in slower tumor growth and a reduction in metastasis^102^.

Other studies have highlighted the existence of lncRNAs that are downregulated in cancer tissues^103^ and the circulation of cancer patients^51^. Such downregulated lincRNAs may be oncosuppressor lncRNAs of which expression is dysregulated during tumorigenesis. The ectopic delivery of synthetic or purified oncosuppressor lncRNAs may constitute a promising therapeutic strategy in the future (Fig. 1). These therapeutic oncosuppressor lncRNAs may be administrated as an exosome-based formula which could possibly treat primary and secondary tumors as it spreads throughout the body via the circulatory system. If some circulating lncRNAs are indeed shown to have oncosuppressive properties in vivo, they may also be uptaken prior to cancer formation for cancer prevention purposes, similar to anti-oxidants (Fig. 1).

### Cancer-specific, multicancer and pan-cancer circulating lncRNA biomarkers and therapeutic targets

**Fig. 2:**
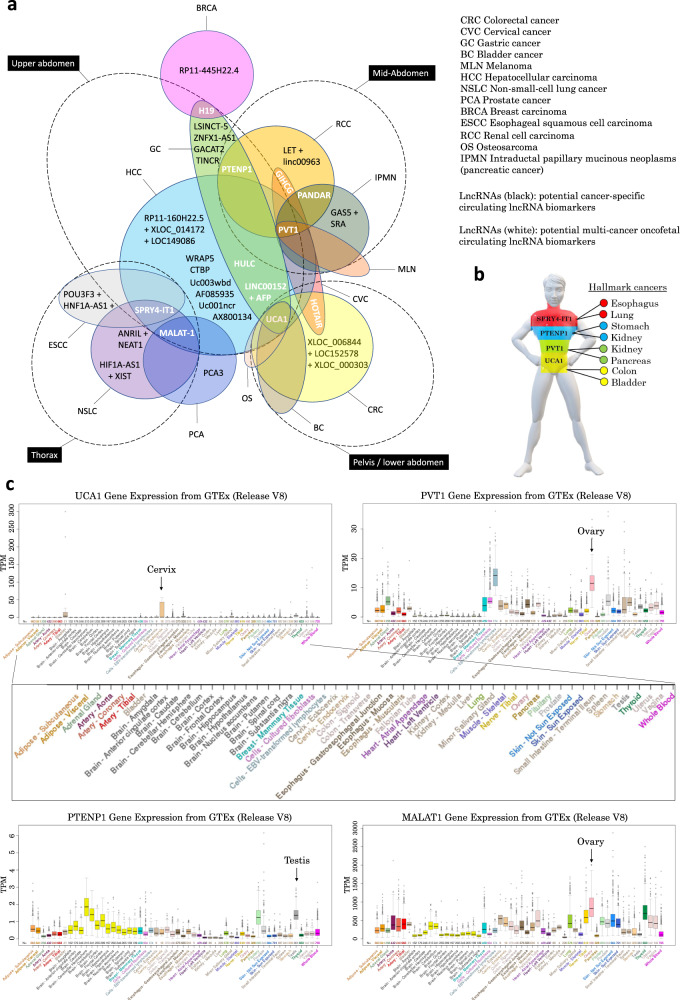
Cancer-specific and multicancer blood-derived lncRNA biomarkers. **a** Diagram showing circulating lncRNAs reported in the literature regrouped by cancer type. Some lncRNAs (in black letters) are cancer-specific. Other circulating lncRNAs (in white letters) such as MALAT1, SPRY4-IT1, PVT1, UCA1 and LINC00152 reflect tumorigenesis in multiple organs. **b** Simplified cartoon representing the specificity of certain circulating lncRNAs towards cancers of organs located in designated anatomic segments of the human body. **c** Gene tissue expression of some of the most widely reported circulating lncRNAs with high multicancer diagnosis potential (GTEx, obtained from UCSC genome browser^188–197^, https://genome.ucsc.edu/).

A significant number of circulating lncRNAs have been reported to be associated with only one cancer type so far (Table 1). While this could be due to a lack of studies on these lncRNAs in other cancer types, it could also imply that certain blood-based lncRNAs may really be specific to a unique type of cancer only, which has significant translational applications especially in cancer screening since the detection of abnormal levels of such lncRNAs in the circulation would not only be indicative of a cancer diagnosis but also pinpoint with accuracy the organ affected by the tumor. More studies need to be undertaken to evaluate the plausibility of these two scenarios. Interestingly, the integrated analysis of the most reported circulating lncRNAs and their specific association with certain cancers seems to reveal a pattern where some circulating lncRNAs are apparently able to reflect multiple cancers especially in organs that are close anatomically and/or embryologically (Fig. 2a, lncRNAs in white letters). For instance, circulating LINC00152, HULC and UCA1 have been associated with gastric and liver cancer, two organs that are in close proximity within the upper abdomen and which both originate from the foregut of the embryonic endoderm^19,30,43,45,46,104^. Lung and esophagus which are located in the thorax and share common embryological origins (before they split apart during development) also show a similar circulating lncRNA - SPRY4-IT1 - upon tumorigenesis^61,63^. Circulating HOTAIR has been detected in the blood of patients with cancers of the uterus and colon/rectum, organs that are located in the pelvis and sometimes fused in congenital diseases such as persistent cloaca^42,105^. Levels of circulating lncRNAs PVT-1 and PANDA reportedly reflect tumorigenesis or malignancy in the kidney and pancreas, two organs that are in close proximity and often grafted together^68,69^. Circulating PVT-1 also reflects tumor formation in the liver, an organ close anatomically and embryologically to the pancreas^64^. The fact that cancers from the same anatomical region or embryological origin display a similar circulating lncRNA molecular signature is consistent with the findings from an integrative study published in 2018 that analyzed the complete set of tumors in The Cancer Genome Atlas (TCGA), consisting of approximately 10,000 specimens and representing 33 cancer types^106^. In this study, the authors performed molecular clustering based on RNA expression levels and other key features and concluded that clustering is primarily organized by histology, tissue type, or anatomic origin^106^. Moreover, the embryological origin of human tumors has been largely discussed and is notably supported by evidence suggesting that adult somatic cells retain an embryonic program that can be reactivated in certain pathological conditions promoting the dedifferentiation into stem cells and eventually tumorigenesis^107^. In addition, machine learning has enabled the identification of key stemness features that are associated with oncogenic dedifferentiation^108^ while embryonic stem cell-like gene expression signatures have been identified in human tumors^109–111^. Because of their involvement in both tumorigenesis and development, several genes including some coding for lncRNAs have been referred to as “oncofetal”^112^. They are reportedly upregulated in the embryo and downregulated in adults^113^. However, in some cancers, these oncofetal lncRNAs may be re-expressed contributing to tumorigenesis and malignancy^114^. In this context, cancer may arise due to loss of cellular differentiation and gain of pluri- or multipotency with the high proliferative potential characteristic of stem cells^115^. This concept notably led to the characterization of cancer stem cells. In fact, it is believed that, as somatic cells from different organs of the same anatomic region dedifferentiate into cancer stem cells, they may indirectly try to recreate the same embryonic organ that was originally responsible for their formation during embryogenesis (which they share in common). Based on this cumulative information, it is perhaps not surprising to observe similar patterns of blood lncRNA levels in cancers with the same embryological or anatomical origin as shown in Fig. 2a, b. However, there are some exceptions and circulating lincRNAs may not necessarily change upon tumorigenesis according to organ location or its embryological origin (e.g. endoderm, mesoderm, ectoderm). For instance, circulating lncRNAs associated with cancer from organs related to reproduction (e.g. prostate, breast) may not follow such an anatomic/embryonic pattern as sexual organs are usually not developed during embryogenesis. Although, in healthy adults, sexual organs appear to be the main sources of some of the most widely reported cancer-associated lncRNAs such as PVT1 and MALAT1 that are mostly expressed in the ovaries of healthy women, while PTENP1 is largely expressed in the testis of healthy men (Fig. 2c). Those lncRNAs mostly remain poorly expressed in other tissues of healthy individuals. The fact that many of these lncRNAs are suppressed in most adult tissues but remain extensively expressed in sexual organs (either ovaries or testis, exclusively) suggests the likely involvement of so-called “genomic imprinting”. It essentially consists in the reprogramming of the epigenetic make-up of certain key genes according to the sex of the individual during gametogenesis, which results in the fetus in a parent-of-origin type of gene expression with transcription occurring only on one allele while being suppressed on the other (notably through DNA methylation and histone modification). *H19* for instance is an imprinted gene that is known to be transcribed exclusively from the maternal allele and silenced on the paternal allele^116^. *H19* is in fact the first imprinted lncRNA-encoding gene ever identified^113^ and its product, the lncRNA H19 (H19 Imprinted Maternally Expressed Transcript), has since been the object of numerous studies to understand its implications in health and disease. H19 lncRNA has notably been reported to play critical roles in both developments^117–119^ and tumorigenesis^120–127^ and therefore legitimately belongs to the class of oncofetal lncRNAs^112,128,129^. A major mechanism by which imprinted lncRNAs such as H19 induce or contribute to tumorigenesis likely involves a still poorly understood event known as “loss-of-imprinting” or LOI that abnormally restores gene expression on both alleles (i.e. “biallelic expression”) in adult somatic cells potentially promoting cancer formation. The reasons for sporadic LOI are not fully understood but likely involve the partial or complete loss of the imprinted epigenetic code of certain key regulatory regions within the DNA sequence notably due to major changes in methylation patterns (e.g. hypomethylation or hypermethylation) that can reportedly be induced by exposure to cigarette smoke for instance. This may affect the ability to recruit insulating proteins such as CTCF resulting in changes in the chromatic structure including de-condensation potentially promoting gene expression on the allele that should otherwise be suppressed. Eventually, it is undeniably clear that circulating imprinted lncRNAs that are expressed during development and which reflect, in adults, tumors from organs with a same embryonic origin could constitute potential “oncofetal imprinted lncRNA biomarkers” as well as promising therapeutic targets. These embryo-derived lncRNAs do represent promising multicancer biomarkers that would not only enable the detection of various types of cancers but also determine the likely location of the tumor in the adult body as well as the organ(s) affected by tumorigenesis. Embryo-related biomarkers such as the carcinoembryonic antigen (CEA) are already in use for the diagnosis of many cancers.

The existence of potential pan-cancer circulating lncRNA biomarkers has also been investigated, including by our lab. Indeed, in a leading study based on the rigorous and systematic statistical analysis of gene expression profiles of twelve different cancer types extracted from multiple publicly available databases, our lab identified 6 promising pan-cancer lincRNA biomarkers subsequently termed “PCAN” lincRNAs that are systematically dysregulated in cancer^103^. Active efforts are currently undertaken to explore the full potential of these PCAN lincRNAs by extending the study to cancers beyond the original 12 cancer types. Upon validation in blood-based samples, this panel of PCAN biomarkers could potentially constitute the first set of circulating lincRNAs capable of detecting any kind of cancer in the human body. Further investigations would also be required to better understand the molecular mechanisms associated with the upregulation of these PCAN lncRNAs in cancer and to assess whether they could constitute potential pan-cancer therapeutic targets as well as imprinted oncofetal genes similar to *H19*.

### Circulating lncRNAs and association with RNA-binding proteins

**Fig. 3:**
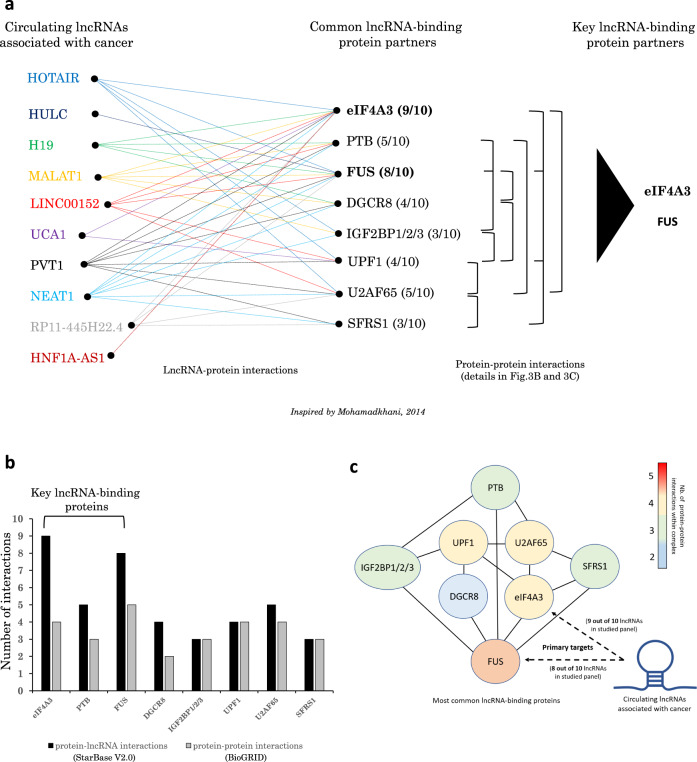
Circulating lincRNAs and a common set of protein partners. **a** Data extracted from starBase V2.0 and lncRNome databases reporting lncRNA-protein interactions occurring in tissues. Indicated lncRNAs share the same set of interacting proteins that are also known to be involved in tumorigenesis. These main proteins may constitute an oncogenic pan-lncRNA core protein interactome. Displayed protein-protein interactions are based on data from BioGRID database. **b** Graph bars representing the number of interactions with lncRNAs and proteins for each RNA-binding protein shown in (**a**). **c** Putative pan-cancer multimeric RNA-binding protein complex showing the different interactions between the proteins that are the most commonly recruited by cancer-related lncRNAs as shown in (**a**).

While RNA-binding proteins may not interact with circulating lncRNAs once they reach the bloodstream, they may bind lncRNAs inside the tumor cells prior to secretion and may actively contribute to the tumorigenic process. Indeed, many RNA-binding proteins that interact with lncRNAs have also been characterized as oncofetal^130,131^. This suggests that lncRNA-related tumorigenesis is likely the result of a complex and diversified molecular mechanism that involves the upregulation of several oncofetal genes, including genes coding for oncofetal lncRNAs and oncofetal lncRNA-binding proteins. Investigators can find information of lncRNA-binding partners by screening databases such as lncRNome, lncRNAMap, starBase V2.0 and UCSC genome browser^132–135^. Further information on the experimental data which support the lncRNA-protein interactions described in Fig. 3 can be found in Table 2. This table provides substantial scientific information that has been extracted from other highly valuable databases such as NPInter^136–139^, BioGRID^140^ and POSTAR3^141^ which rigorously report data from Affinity Capture-Mass Spectrometry (BioGRID terminology)^142^, UV Cross-Linking and Immunoprecipitation (CLIP) / CLIP-seq / HITS-CLIP^143–149^, Photoactivatable Ribonucleoside-enhanced Crosslinking and Immunoprecipitation (PAR-CLIP)^150–153^, Enhanced CLIP (eCLIP)^154,155^, Individual-nucleotide resolution UV Crosslinking and Immunoprecipitation (iCLIP), Capture Hybridization Analysis of RNA Targets (CHART-seq)^156^, Affinity Chromatography^157^, as well as other methods such as RNA Immunoprecipitation (RIP), Affinity Capture-RNA (BioGRID terminology)^158–161^ and other “Protein-RNA” methods (BioGRID terminology)^162–164^ which may also include a combination of Immunocytochemistry (ICC), In Situ Hybridization, Northern Blot and/or RT-PCR^165,166^.

**Table 2:** Experimental data supporting interactions between lncRNAs and RNA-binding proteins (RBPs) that are commonly associated with cancer.

LncRNAs commonly associated with cancer									
Most common RNA-binding proteins (RBPs)	HOTAIR	HULC	H19	MALAT1	LINC00152 (CYTOR)	UCA1	PVT1	NEAT1	HNF1A-AS1
eIF4A3			CLIP-seq / HITS-CLIP (NPInter, POSTAR3)^143^	CLIP-Seq / HITS-CLIP; iCLIP (NPInter, POSTAR3)^143^			CLIP-seq / HITS-CLIP; iCLIP (NPInter, POSTAR3)^143^	CLIP-Seq / HITS-CLIP (NPInter, POSTAR3)^143^	
PTB (PTBP1)	iCLIP (POSTAR3)	Affinity Capture Mass Spectrometry (BioGRID)^142^	PAR-CLIP; HITS-CLIP (NPInter, lncRNome, POSTAR3)	PAR-CLIP; HITS-CLIP; iCLIP (NPInter, lncRNome, POSTAR3)	PAR-CLIP; HITS-CLIP; iCLIP; eCLIP (NPInter, lncRNome, POSTAR3)	eCLIP (POSTAR3)	PAR-CLIP; HITS-CLIP; iCLIP (NPInter, lncRNome, POSTAR3)	PAR-CLIP; HITS-CLIP; iCLIP (NPInter, lncRNome, POSTAR3)	
FUS		CLIP-seq (NPInter)^144^	CLIP-Seq (NPInter)^144^	,CLIP-Seq; PAR-CLIP (NPInter, POSTAR3)^150,144,153^	CLIP-seq / HITS-CLIP; eCLIP (NPInter, POSTAR3)^144^		CLIP-Seq; PAR-CLIP (NPInter, POSTAR3)^150,144^	,RIP; CHART-seq CLIP-Seq; PAR-CLIP (NPInter, POSTAR3)^150,144,145,156^	PAR-CLIP (POSTAR3)
DGCR8			eCLIP (NPInter, POSTAR3)^154^	eCLIP; HITS-CLIP (NPInter, POSTAR3)^154^	eCLIP (NPInter)^154^	eCLIP (POSTAR3)	eCLIP (NPInter)^154^	,eCLIP; CHART-seq; HITS-CLIP (NPInter, POSTAR3)^154,156^	eCLIP (NPInter)^154^
IGF2BP1/2/3	PAR-CLIP (NPInter, lncRNome, POSTAR3)^151^	Affinity Chromatography (NPInter)^157^	,iCLIP; PAR-CLIP; RT-PCR In situ Hybridization Northern Blot (NPInter, POSTAR3)^165,166^	,eCLIP; PAR-CLIP; iCLIP (NPInter, lncRNome, POSTAR3)^151,154,155^	PAR-CLIP (POSTAR3)	iCLIP; eCLIP (POSTAR3)	PAR-CLIP; eCLIP; iCLIP (NPInter, lncRNome, POSTAR3)^154^	,eCLIP; PAR-CLIP; iCLIP; CHART-Seq (NPInter, POSTAR3)^151,154–156^	eCLIP (NPInter)^154^
UPF1	PAR-CLIP; HITS-CLIP (NPInter, POSTAR3)^152^		HITS-CLIP (POSTAR3)	,eCLIP; PAR-CLIP; HITS-CLIP; iCLIP (NPInter, POSTAR3)^154,146,152^	eCLIP; HITS-CLIP; iCLIP (NPInter, POSTAR3)^154^	iCLIP (POSTAR3)	,eCLIP; PAR-CLIP; iCLIP; HITS-CLIP (NPInter, POSTAR3)^154,146,152^	,eCLIP; PAR-CLIP; HITS-CLIP (NPInter, POSTAR3)^154,146,152^	PAR-CLIP (NPInter)^152^
U2AF65	iCLIP (POSTAR3)		iCLIP (POSTAR3)	iCLIP (POSTAR3)	iCLIP (POSTAR3)			iCLIP (POSTAR3)	
SFRS1	iCLIP (POSTAR3)		PAR-CLIP; iCLIP (POSTAR3)	,iCLIP; Microarray eCLIP; CLIP-Seq CHART-seq; PAR-CLIP (NPInter, POSTAR3)^147–149,154,156^	eCLIP; iCLIP (NPInter, POSTAR3)^154^		,eCLIP; CLIP-Seq; PAR-CLIP; iCLIP (NPInter, POSTAR3)^147,154^	,eCLIP; CLIP; PAR-CLIP CLIP-Seq; CHART-seq (NPInter, POSTAR3)^147,154,156^	eCLIP (NPInter)^154^
Other RBPs of interest	,AGO2, ELAVL1, EZH2. Affinity Capture-RNA; Protein-RNA (BioGRID, POSTAR3)^160–164^		AGO2. PAR-CLIP; HITS-CLIP Affinity Capture - RNA (POSTAR3, BioGRID)^158^	AGO2, ELAVL1, EZH2. PAR-CLIP; HITS-CLIP; iCLIP Affinity Capture - RNA (POSTAR3, BioGRID)^158^	AGO2, ELAVL1. PAR-CLIP; HITS-CLIP; iCLIP (POSTAR3)		AGO2, ELAVL1. PAR-CLIP; HITS-CLIP (POSTAR3)	AGO2, ELAVL1. PAR-CLIP; HITS-CLIP; iCLIP (POSTAR3)	AGO2, ELAVL1. PAR-CLIP Affinity Capture - RNA (POSTAR3, BioGRID)^159^

Information extracted from several databases including NPInter^136–139^, BioGRID^140^, lncRNome^134^ and POSTAR3^141^. *CLIP* UV Cross-Linking and Immunoprecipitation, *PAR-CLIP* Photoactivatable Ribonucleoside-enhanced Crosslinking and Immunoprecipitation, *eCLIP* Enhanced CLIP, *iCLIP* Individual-nucleotide resolution UV Crosslinking and Immunoprecipitation, *CHART-seq* Capture Hybridization Analysis of RNA Targets, *RIP* RNA Immunoprecipitation.

**Table 3:** Guidelines recommended for the study of circulating lncRNAs as biomarkers for cancer diagnosis, based on troubleshooting performed by previous works.

Step	Recommended	Reason	Reference
Patient selection	Exclude patients with inflammation	Higher / different levels of white blood cells associated with inflammation may impact levels of circulating RNAs upon cytolysis	^198,199^
	Recruit patients with same gender, age and race	Minimize variation in lncRNA levels due to possible inter-individual confounding factors (such as SNPs, CNV, etc.)	^63^
	May include questionnaire about diet and lifestyle	Diet and lifestyle (alcohol consumption, smoking) can affect lncRNA levels	^200,201^
Blood sample preparation	Prepare serum or plasma. Discard cellular fraction	Cellular fraction of blood may contain different levels of blood cells which in return may impact levels of circulating RNAs upon cytolysis	^199,202^
	Strict standard operating procedures when preparing serum/plasma	Minimize variations in circulating RNAs due to sample preparation. Avoid hemolysis.	^202^
	Measure A_414_, A_541_, A_576_	Assess for hemolyzed samples	^69^
RNA extraction	Use kits compatible with liquid samples	Enable extraction of circulating lncRNAs from plasma or serum samples	Kit manufacturers
	Use kits combining both solid (filter) and liquid phase (organic) extraction	Maximize extraction of circulating lncRNAs from plasma or serum samples	^17,24,42^
	Use as much plasma/serum as possible	Maximize RNA yield after extraction	Our recommendation
Reverse Transcription	Use same volume of RNA extracts	Allow maximum RNA input for Reverse Transcription	Our recommendation
qPCR (relative quantification with ΔΔCt method)	Test several reference genes. Carefully choose best reference gene(s) using NormFinder, RefFinder or Genorm algorithms. Most popular: GAPDH, beta-actin, 18 S To avoid: RPLPO, GUSB, HPRT	The right reference gene is needed for accurate relative quantification using ΔΔCt method. GAPDH, beta-actin, 18 S present in large quantities in blood. RPLPO levels inconsistent in blood GUSB, HPRT levels too low in blood	^3,25,41,42,47,203^
	Careful in interpretation of data when using spike-in controls	Spike-in controls do not account for variations in lncRNA concentrations in blood-derived samples prior to RNA extraction step	^180^
	Measure transcript levels of *MB, NGB, CYGB genes*	Assess for contamination from red blood cells	^69^
	Measure transcript levels of *APOE, CD68, CD2, CD3 genes*	Assess for contamination from white blood cells	^69^

Information reported includes step of the analysis, actual recommendation, reason for the recommendation and related literature reference.

**Fig. 4:**
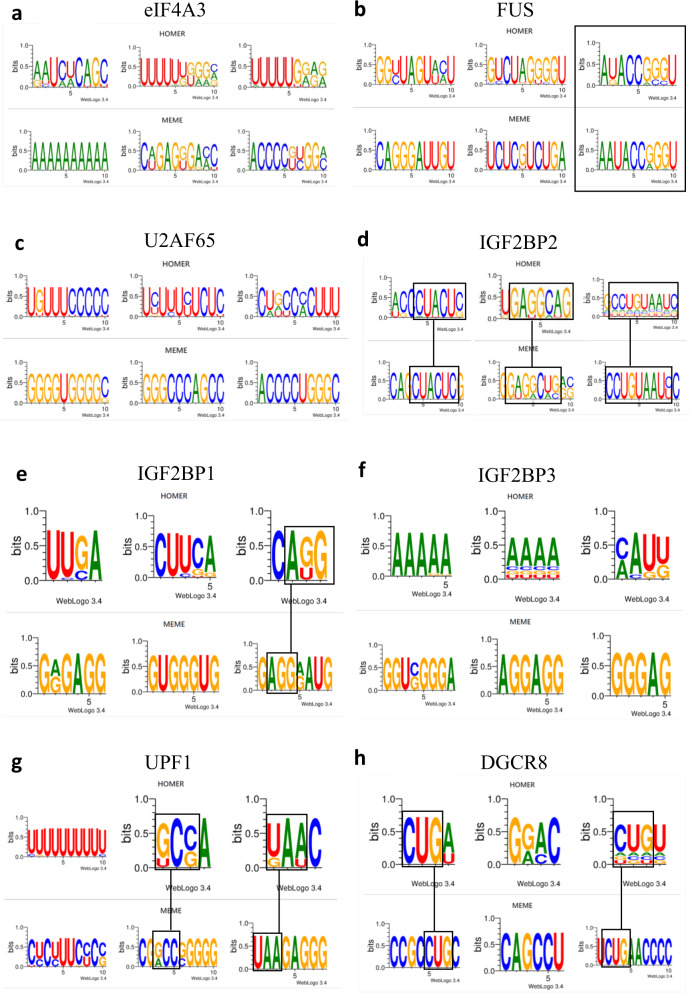
Putative consensus motifs in lncRNAs for the specific binding of key RNA-binding proteins. Data extracted from POSTAR3 database (CLIPseq-based)^141^ and processed by HOMER and MEME algorithms that are commonly used for motif discovery and next-generation sequencing (NGS) data analysis. Square boxes highlight similar patterns identified in the motifs provided by both algorithms. **a** Consensus motif for binding of RNA-binding protein eIF4A3 (eukaryotic initiation factor 4A-III). **b** Consensus motif for binding of RNA-binding protein FUS (fused in sarcoma). **c** Consensus motif for binding of RNA-binding protein U2AF65 (splicing factor U2AF 65kDa subunit). **d** Consensus motif for binding of RNA-binding protein IGF2BP2 (insulin-like growth factor 2 mRNA-binding protein 2). **e** Consensus motif for binding of RNA-binding protein IGF2BP1 (insulin-like growth factor 2 mRNA-binding protein 1). **f** Consensus motif for binding of RNA-binding protein IGF2BP3 (insulin-like growth factor 2 mRNA-binding protein 3). **g** Consensus motif for binding of RNA-binding protein UPF1 (regulator of nonsense transcripts 1). **h** Consensus motif for binding of RNA-binding protein DGCR8 (microprocessor complex subunit DGCR8, DiGeorge syndrome critical region 8).

Systematic analysis of these databases actually revealed a common set of proteins that consistently interacts with the most reported cancer-related lncRNAs (Fig. 3a)^167^. Most of these proteins are associated with cancer formation upon dysregulation, especially IGF2BP3^168,169^, FUS^170,171^ and eIF4A3^172^. This suggests the likely existence of a pan-lincRNA core protein interactome that may, by itself, be sufficient to promote tumorigenesis. However, some of these proteins appear to be more frequently involved in lncRNA interactions than others and may play a more central role in cancer formation. For instance, eIF4A3 was found to interact with 9 of out 10 lncRNAs in the lncRNA panel reported here (Fig. 3a, b), while FUS was recruited by 8 out of 10 lncRNAs. Therefore, eIF4A3 and FUS may constitute key lncRNA-binding proteins that could be part of a pan-cancer molecular mechanism that mediates the tumorigenic properties of most oncogenic lncRNAs and/or generally promotes lncRNA secretion into the systemic circulation from the tumor site. Thus, eIF4A3 and FUS may represent major pan-cancer therapeutic targets. While other RNA-binding proteins appear to be less frequently recruited by cancer-related lncRNAs, they may still exert pan-tumorigenic properties since all RNA-binding proteins reported here in Fig. 3a are part of a very same multimeric protein complex based on data from an extensive search of protein-protein interactions using BioGRID database (Fig. 3c). Interestingly, eIF4A3 and FUS showed the highest ability to interact with other RNA-binding proteins (respectively binding 4 and 5 other protein partners within the complex), which may explain why they are often associated with lncRNAs since the more lncRNA-binding proteins they bind, the more lncRNAs they collect. Given the relatively high frequency of recruitment of eIF4A3, FUS and related RNA-binding proteins (RBPs) by cancer-associated lncRNAs and their known roles in tumorigenesis, we here provide in Fig. 4 the putative consensus motifs that enable lncRNAs to specifically bind these RBPs, as this may help investigators to identify novel interactions between their lncRNA of interest and these tumorigenic RBPs (consensus motifs extracted from POSTAR3 database which reports CLIP-seq data^141^).

Overall, it is clear that lncRNAs and their interacting partners will constitute innovative therapeutic targets and/or agents in future cancer therapy strategies.

## Discussion and future perspectives

Circulating lncRNAs have been shown to constitute reliable biomarkers for both cancer diagnosis and prognosis. They have also been suggested as potential therapeutic targets, notably due to the fact that they are reportedly transported in the bloodstream by exosomes which are known to contribute to cancer progression and metastasis by enabling communication between cancer cells that produce those exosomes and non-cancerous “target” cells which may be incited to transform into new cancer cells under exposure to exosome-borne oncogenic lncRNAs^99^. Interestingly, those tumor-derived exosomes (or TD-exosomes) appear to display a unique molecular signature that differs from that of non-cancerous exosomes potentially providing a window of opportunity for future antitumoral therapies aiming to stop the formation of secondary tumors by specifically targeting TD-exosomes. In terms of diagnostic performance, while it can be improved by combining multiple lncRNAs, it is important to note that the “specificity” determined in the reported studies refers to the comparative analysis of samples from healthy volunteers and patients with specific cancer. In this particular context, “specificity” does not describe the ability to distinguish a certain cancer type from other cancers. This is particularly relevant since several circulating lncRNAs have been proposed as potential biomarkers for a large variety of different cancers. For instance, MALAT-1 could be used to diagnose prostate cancer^26^ and nonsmall-cell lung cancer^25,51^. Similarly, HOTAIR has the potential to detect both colorectal^42^ and cervical cancer^105^. LINC00152 could lead to the diagnosis of both hepatocellular carcinoma^43^ and gastric cancer^19^. LncRNA GIHCG has been shown to be involved in the pathogenesis of many types of different cancers including liver, cervical, gastric, renal and colorectal cancer for which it may constitute a promising biomarker^33,34,90,173–175^. PVT1 has been reported as a potential circulating biomarker (alone or in combination with other lncRNAs) for at least five different types of cancers including RCC (kidney), IPMN (pancreas), HCC (liver), MLN (skin), and CVC (cervix)^64,68,176,177^. UCA1 constitutes another lncRNA with significant multicancer diagnostic potential since it has been reported to effectively detect (alone or in combination with other lncRNAs) at least five distinct cancers such as HCC (liver), GC (stomach), BC (bladder), CRC (colon) and osteosarcoma (bone)^41,45,46,104,178,179^.

The increasing number of studies on circulating lincRNAs may eventually indicate that all circulating lncRNAs reflect more than one cancer and that there is no unique biomarker for each cancer type or subtype. It has especially been suggested that changes in lncRNA level in the circulation of cancer patients could be due to a general pathophysiological response from the body to the presence of tumors and not due to direct secretions from the tumors themselves^180^. This represents a strong argument as significant levels of lncRNAs have been detected in the blood of cancer-free healthy subjects. This would also explain why there is sometimes a lack of correlation between circulating lncRNA levels and cancer tissue lncRNA levels. Thus, circulating lncRNAs may actually reflect the presence of tumors in general. In this context, it is likely that in the near future pan-cancer circulating biomarkers could be identified. On the other hand, the findings from recent studies suggest that the detection of a specific cancer type may be achieved by using multi-analyte liquid biopsy and multi-modal strategies, including lncRNA detection^181,182^. For instance, to better predict specific lncRNA-cancer associations, Yan et al. developed an original method termed DRACA (for “detecting lncRNA-cancer association”), based on the analysis of five different types of features including lncRNAs, miRNAs, genes, cancer types and cancer prognosis (3)^181^. We here provide the name of the databases used by the authors, as these may be useful to other investigators. StarBase v2.0 was used for lncRNA–miRNA relationships^135^, lncReg for lncRNA–gene interactions^183^, lncRNADisease for lncRNA–cancer associations^184^, miRTarbase for miRNA–gene relationships^185^, MNDR v2.0 for miRNA–cancer relationships^186^ and DisGeNet for gene–cancer relationships^187^. DRACA eventually outperformed other methods in predicting specific lncRNA-cancer associations^181^. In another outstanding study, Sanchez-Salcedo et al. reported that the specific detection of prostate cancer can be performed by using a dual electrochemical hybridization-based biosensor with enzymatic signal amplification for the detection of both PCA3 lncRNA and PSA mRNA (prostate-specific antigen, non-lncRNA)^182^. One major advantage of this technique compared to commercial tests, is that it reportedly enables the detection of PCA3 lncRNA in urine samples of prostate cancer patients without prior RNA amplification. Because the study of circulating lncRNAs via traditional RT-qPCR or next-generation sequencing methods can sometimes be quite challenging, we here provide relevant guidelines that may be useful to investigators who are new to the field (boxes 1–4, and Table 3).

Overall, while the study of circulating lncRNAs is still at an early stage, the worldwide growing interest in lncRNAs and the emergence of new technologies to improve their detection, specificity, and potential in clinical applications undeniably increases the chance of discovering one day reliable blood-based biomarkers that will allow the early and accurate detection of any type of cancer.

Box 1 Advice on patient recruitment and sample selection when studying circulating RNAs as biomarkers for early cancer diagnosis

- While whole blood has been successfully used in circulating lncRNA studies^51^, it is usually not recommended for accurate quantification of circulating RNAs due to variability associated with red and white blood cells^202^. Indeed, levels of white blood cells (and thus circulating RNAs) are likely to change if patients are experiencing chronic or acute inflammation which may not necessarily be related to the disease investigated^198,199^. Cell-free samples such as plasma (blood fraction obtained with anti-coagulants) and serum (blood fraction obtained after coagulation) are more reliable sources of circulating lncRNAs and have been largely used in studies comparing circulating lncRNA levels in cancer patients and healthy subjects (Table 1).
- Levels of circulating RNAs may also vary within the same group of individuals (e.g. healthy volunteers) due to internal factors such as patient hydration level or diet^200,201^ as well as age, gender and race. Copy number variations (CNVs) and single nucleotide polymorphisms (SNPs) have also been proposed as possible sources of variations in levels of circulating lncRNAs. Consequently, investigators usually collect relevant patient information and compare individuals with similar records.
- Equal volumes of plasma from different patients may not contain the same RNA concentration. Inconsistent serum or plasma preparation across samples may add another level of variability in RNA content especially if hemolysis could not be avoided. To account for hemolysis, Permuth et al. visually inspected their samples and measured absorbance at three different wavelengths. An absorbance exceeding 0.2 for any of these wavelengths indicated hemolyzed samples^69^. They further assessed for blood-cell contaminants by measuring levels of transcripts from *MB*, *NGB* and *CYGB* genes (for erythrocytes) as well as *APOE, CD68*, *CD2* and *CD3* (for leucocytes).

Box 2 Extraction of circulating lncRNAs from liquid biopsy: Pitfalls and Recommendations

- Investigators that wish to use column-based kits should be aware that most commercially available kits are optimized for non-liquid samples such as cells or tissues, and not for plasma or serum. Some kits such as the miRNeasy Serum/ Plasma kit do allow RNA extraction from serum and plasma, but it is mostly designed for purification of microRNAs (miRNAs) and other small RNAs.
- Since lncRNAs are naturally scarce in circulation, investigators may wish to use large volumes of plasma or serum to increase the RNA yield upon extraction. However, most kits are provided with columns of limited size which may introduce variability in RNA yields, as investigators often have to perform successive column-based purifications with small volumes of the same sample. If different kit formats are available (for instance mini, midi and maxi), investigators should proceed with the kit that is the most suitable for their study based on the volume of samples that is available to them. Note that if the volume of the original plasma sample is too small, the RNA yield might be too low for RNA quantification and qPCR detection.
- Despite the relatively low RNA yield generated from blood-based samples, most kits provide RNA samples of high purity due to solid-phase extraction and multiple washing steps. Improved RNA extraction may come from the addition of an organic extraction based on liquid phase separation using phenol/chloroform. For instance, the mirVana kit which combines both solid phase (filter) and liquid phase (chloroform) RNA extraction has been largely used in cancer studies focusing on circulating lncRNAs^17,26,42^. This kit appears popular among investigators because it allows total RNA extraction from liquid samples (plasma/serum) as well as purification of small RNAs and lncRNAs.

Box 3 Potential issues when performing RT-qPCR with circulating lncRNAs from liquid biopsy

- Investigators have the choice to use equal amounts of total RNAs or equal volumes of RNA extracts.
- Using equal quantities of total RNAs has drawbacks since the quantity of total RNAs obtained after extraction from plasma or serum samples is usually very low and may be undetectable using spectrophotometers, while the adjustment of all samples to the sample with the lowest RNA concentration may reduce the output of the subsequent qPCR reaction.
- When using equal volumes of RNA extracts, normalization using reference genes becomes indispensable for the analysis of qPCR data by relative quantification (ΔΔCt method). However, there is no consensus on the best reference genes. They are case-sensitive and must be evaluated for each study, which may be challenging due to inherent variability associated with cancer^199,202^. Ultimately, one must determine whether these variations are statistically significant or not^25^. Usually, the least variable candidate is selected by using algorithms such as NormFinder, RefFinder or Genorm^25,41,42^.
- Due to poor abundance or recurrent variability, several genes should be avoided. *RPLPO* which is a commonly used for tissue sample analysis is not recommended for the quantification of blood-based biomarkers^25,203^, while HPRT and GUSB transcripts are not abundant enough in normal human serum^41^.
- Exogenous spike-in controls may be used to account for variability introduced during RNA extraction, however they do not reflect inherent variations in RNA concentrations prior to RNA extraction^180^. We recommend using both spike-in controls and reference genes to better account for variations in circulating lncRNA levels. Investigators may also evaluate lncRNA levels by absolute qPCR quantification using standard curves made with reference standards^38^.
- All guidelines from Boxes 1, 2 and 3 are summarized in Table 3.

Box 4 Tips for analyzing circulating lncRNAs with next-generation sequencing-based technologies

- There are two paradigms in next-generation sequencing technology: short-read sequencing (35–700 bp)^204^ and long-read sequencing (>5000 bp)^205^. Short-read sequencing provide lower-cost, higher-accuracy data that are useful for population-level research and clinical variant discovery^204^, while long-read approaches provide read lengths that are well suited for de novo genome assembly applications and full-length isoform sequencing^205^. In practice, short-read sequencing is usually used for cancer early diagnosis^206^.
- It is necessary to enhance lncRNA concentration by building lncRNA-specific cDNA library using oligonucleotide capture technology^207^. With complementary oligonucleotide probes, this technology increases the concentration of lncRNA sequences by at least 25%^207^. The optimization of probe sequences is similar to that for microarray technologies^208^.
- The computational preprocessing of lncRNA sequencing requires using Quality-Control (QC) tools such as FastQC^209,210^ or AfterQC^211^. The processed reads are then aligned to a noncoding transcriptome reference such as LNCipedia^212^, using tools such as HISAT2^213^ or BowTie2^214^. The mapped reads are assembled to lncRNAs using tools such as StringTie2^215^. The quantity of lncRNAs is normalized by the sequencing depth and transcript length using RPKM, FPKM or TPM method^216^. The differentially expressed lncRNAs are detected by tools such as DESeq2^217^ or edgeR^218^. Risk score analysis can determine the association between cancer and differentially expressed lncRNAs^219^. By associating lncRNAs with the nearest protein coding genes, their biological functions may be explored through gene ontology (GO)^220^ and KEGG pathway^221^ analyses. Finally, classification models can be used to identify potential lncRNA biomarkers, with individual training, testing and valiation datasets^222,223^. Metrics such as AUC in ROC are used to measure the effectiveness of the lncRNAs in predicting cancer^103^.

### Reporting Summary

Further information on research design is available in the Nature Research Reporting Summary linked to this article.

## Supplementary information

REPORTING SUMMARY

## Data Availability

Weblinks to publicly available databases mentioned in this manuscript are provided below. LncRNA-protein interactions: NPInter: http://bigdata.ibp.ac.cn/npinter4/ POSTAR3: http://POSTAR.ncrnalab.org/ StarBase: https://starbase.sysu.edu.cn/starbase2/ lncRNome: http://genome.igib.res.in/lncRNome/ Protein-protein interactions: BioGRID: https://thebiogrid.org/ LncRNA expression (GTEx): UCSC Genome Browser ^188–197^: https://genome.ucsc.edu/ 3D models obtained from Paint 3D (Microsoft, https://www.microsoft.com/en-us/) and used with the permission from Microsoft. The data generated and/or analyzed in the current study are available from the corresponding author on reasonable request. All data generated or analyzed during this study are included in this published article.
